# Restriction of protein synthesis abolishes senescence features at cellular and organismal levels

**DOI:** 10.1038/srep18722

**Published:** 2016-01-05

**Authors:** Yuki Takauji, Takumi Wada, Asuka Takeda, Ikuru Kudo, Kensuke Miki, Michihiko Fujii, Dai Ayusawa

**Affiliations:** 1 Graduate School of Nanobioscience, Yokohama City University, 22-2 Seto, Kanazawa-ku, Yokohama, Kanagawa 236-0027, Japan; 2 Ichiban Life Corporation, 1-1-7 Horai-cho, Naka-ku, Yokohama, Kanagawa 231-0048, Japan

## Abstract

Cellular senescence or its equivalence is induced by treatment of cells with an appropriate inducer of senescence in various cell types. Mild restriction of cytoplasmic protein synthesis prevented induction of all aspects of cellular senescence in normal and tumor-derived human cells. It allowed the cells to continuously grow with no sign of senescent features in the presence of various inducers. It also delayed replicative senescence in normal human fibroblasts. Moreover, it allowed for growth of the cells that had entered a senescent state. When adult worms of the nematode *C. elegans* were grown under protein-restricted conditions, their average and maximal lifespans were significantly extended. These results suggest that accumulation of cytoplasmic proteins due to imbalance in macromolecule synthesis is a fundamental cause of cellular senescence.

Cellular senescence constitutes a major determinant of lifespan in animal models, and can largely account for dysfunctions due to ageing. Senescence occurs with appropriate inducers in virtually every cell type. During senescence, normal human cells exhibit irregularly enlarged and flat cell shape, and up-regulate particular genes called the senescence-associated genes, and eventually lose division potential^1^,^2^. Immortalized or tumor cell lines also show similar phenomena by treatment with appropriate inducers^3^. In view of common features of senescent cells, we have postulated that senescence features simply represent a terminal phenomenon of unbalanced growth^4^. While senescent features differ considerably depending on experimental systems, retardation in DNA replication and cell swelling are commonly observed. These observations suggest that cellular senescence is a terminal phenomenon of unbalanced growth^4^.

During unbalanced growth, DNA replication proceeds slowly with RNA and protein synthesis not altered^5^, resulting in an increase in protein content per cell^5^. Morphologically, enormous cell swelling and nuclear swelling occur^6^. Genetically, particular genes defined as the senescence-associated genes that are located preferentially on the heterochromatin are expressed^7^,^8^. Since protein is the predominant form of macromolecules in a cell, accumulation of membrane-impermeant proteins in cells gives rise to osmotic pressure within the cell^9^. In fact, the contents of DNA, RNA, and protein are shown to be approximately 10, 30, and 900 pg per cell, respectively, in cervical tumor-derived HeLa cells^10^. This accumulation of protein in a cell generates influxes of water and/or ions across the water-permeable plasma membrane, and results in cell swelling^11^.

We have observed that cell swelling is linked with nuclear swelling, possibly leading to disintegration of the nuclear envelope. This can account for expression of the senescence-associated genes located on the heterochromatin^7^,^8^. Since the heterochromatin and nuclear envelope form a complex, disruption of the nuclear envelope will decondense the heterochromatin, a state necessary to express the genes 8. In fact, we have observed that these phenomena are always linked with senescence in our systems^3^,^4^,^6^,^7^,^8^.

In our previous studies, we tried to suppress cellular senescence in several systems^4^,^12^. Consequently, we have found that when HeLa cells and TIG-7 normal human fibroblasts were grown in normal medium in the presence of high concentration (3 μM) of cycloheximide, it prevented the lethal effects of excess thymidine and aphidicolin^4^,^12^. Temporary addition of the inhibitors of ERK1/2 such as U0126 was also found to suppress induction of senescence in HeLa but not in TIG-7 cells with reduction in cell volume although an increase in protein content per cell was not reduced^4^,^12^.

In this study, based on the above results, we tried to completely suppress the senescence features at the cellular and organismal levels. Consequently, we were able to demonstrate that mild restriction of cytoplasmic protein synthesis abolishes all aspects of induced and replicative senescence^13^ in virtually every cell type and extends lifespan in the nematode *C. elegans*.

## Results

### Effect of inhibition of protein synthesis on colony formation

First, we searched for an inhibitor of protein synthesis to suppress cellular senescence. Senescence was induced in HeLa and TIG-7 cells by treatment with excess thymidine, an inducer of pool imbalance of DNA precursors, camptothecin, an inhibitor of DNA topoisomerase I, and aphidicolin, an inhibitor of DNA polymerase α^4^,^14^. These inducers were all found to induce typical senescence features coupled with retardation in DNA replication in virtually every cell type^4^. Upon addition of such inducers, the cells gradually stopped dividing, and exhibited enlarged, flat cell shape and clear staining patterns with the senescence-associated ß-galactosidase. Such cells gradually lost division potential, and thus formed tiny colonies.

**Figure 1:**
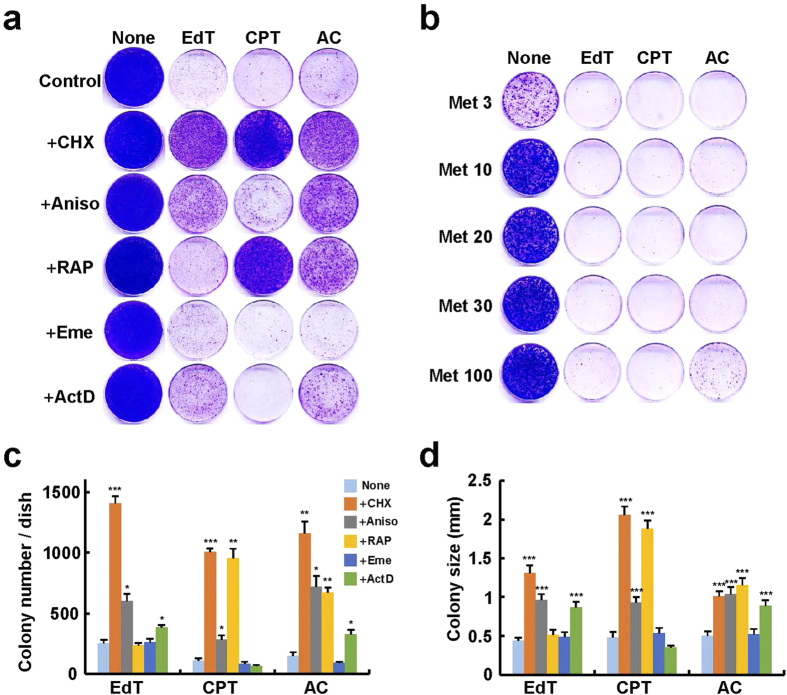
Effect of inhibition of protein synthesis on the loss of colony forming ability induced by several agents in HeLa cells. (**a**) Cells were cultured in the presence of the agents together with the inhibitors of protein synthesis indicated for 2 weeks, and colonies formed were stained with Coomassie Brilliant Blue. None, no addition; EdT, 2 mM excess thymidine; CPT, 7.5 nM camptothecin; AC, 0.3 μM aphidicolin; + CHX, 0.15 μM cycloheximide; + Aniso, 0.057 μM anisomycin; + RAP, 0.055 μM rapamycin; + Eme, 0.033 μM emetine; + ActD, 0.8 nM actinomycin D. (**b**) Cells were cultured under methionine-restricted conditions for 2 weeks, and processed as in (**a**). None, no addition; Met3-Met100, 3–100 mg methionine/L. (**c**) Quantification of the colony numbers in (a) (n = 3). (**d**) Quantification of the colony sizes in (**a**) (n = 50). Colony sizes were measured for 50 colonies. Bars denote means with a standard error of the mean (SEM). *P < 0.05, **P < 0.01 and ***P < 0.001 in comparison with the cells added with the agent.

We used various inhibitors of protein synthesis with a mechanism different from each other at a wide range of concentrations. The inhibitors were added to HeLa cells together with the above inducers, and the number and size of colonies formed were observed as a criterion for suppression of senescence (Fig. 1a,c,d). Simultaneous addition of very low concentrations (e.g., 0.1 μM) of cycloheximide, which blocks the translational elongation step in cytoplasmic but not mitochondrial protein synthesis^15^, was found to clearly suppress senescence induced by excess thymidine, camptothecin, or aphidicolin in HeLa cells. Anisomycin, which inhibits peptidyl transferase or the 80S ribosome system with strong side effects^16^, suppressed the loss of colony forming abilities induced by the above agents, although its effects were less pronounced than those of cycloheximide. Rapamycin, which inhibits the activity of p70 S6 kinase, synthesis of elongation factor 2, and polysomal association of mRNAs^17^, significantly suppressed the loss of colony forming abilities induced by camptothecin or aphidicolin. However, rapamycin stimulated cell death when added with excess thymidine. This may be due to inhibition of mismatch repair by rapamycin^18^ because excess thymidine induces expansion of the dTTP pool, resulting in misincorporation of T instead of C during DNA replication^19^. Emetine, which inhibits both cytoplasmic and mitochondrial protein synthesis^20^, did not significantly suppress the loss of colony forming abilities in any case. Actinomycin D, which inhibits nuclear and mitochondrial ribosomal RNA synthesis^21^, weakly but significantly suppressed the loss of colony forming abilities induced by excess thymidine or aphidicolin, but not in the case of camptothecin. Methionine restriction was not effective at a wide range of concentrations although its positive effect on longevity has been reported in normal human cells^22^ (Fig. 1b). These results suggest that mild restriction of cytoplasmic protein synthesis can suppress the loss of colony forming ability induced by the typical inducers of senescence.

### Effect of cycloheximide on cellular senescence features

**Figure 2:**
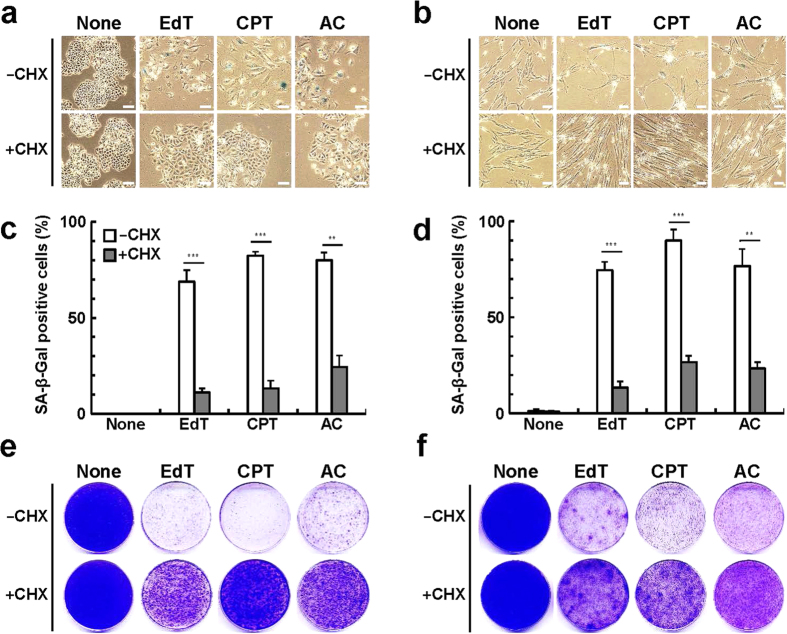
Effect of cycloheximide on senescence features induced by several agents in human cells. (**a**) HeLa cells were cultured in the presence of the agent indicated for 2 weeks, stained with the senescence-associated ß-galactosidase, and subjected to photography. None, no addition; CHX, 0.15 μM cycloheximide; EdT, 2 mM thymidine; CPT, 7.5 nM camptothecin; AC, 0.3 μM aphidicolin. (**b**) TIG-7 cells were cultured in the presence of the agent indicated for 3 weeks, and processed as in (**a**). None, no addition; CHX, 0.15 μM cycloheximide; EdT, excess 1 mM thymidine; CPT, 2 nM camptothecin; AC, 0.2 μM aphidicolin. (**c**) Numbers of HeLa cells stained with the senescence-associated ß-galactosidase were calculated (n = 3). (**d**) Numbers of TIG-7 cells stained with the senescence-associated ß-galactosidase were calculated (n = 3) as in (**c**). (**e**) Colonies of HeLa cells were stained with Coomassie Brilliant Blue. (**f**) Colonies of TIG-7 cells were stained with Coomassie Brilliant Blue. Bars denote means with a standard error of the mean (SEM). **P < 0.01 and ***P < 0.001, respectively, in comparison between -CHX and + CHX. Scale bars, 100 μm.

We examined the effects of the low concentrations of cycloheximide on several senescence features induced by the above agents in HeLa and TIG-7 cells. The effective concentration of cycloheximide itself had no effect on growth in both of HeLa and TIG-7 cells as judged by their morphology and numbers and sizes of colonies (Fig. 2). In the presence of any of the inducers, both types of cells exhibited the typical senescent cell shape and were clearly stained with the senescence-associated ß-galactosidase (Fig. 2a–d). Then we assessed whether restriction of protein synthesis can allow for continuous growth of the cells cultured with the above inducers. Upon addition of the inducers, the cells stopped growing after culture for 5–6 days in both cell types. When HeLa cells were cultures in the presence of camptothecin for 7 days, they accumulated in G_2_/M phase of the cell cycle (Supplementary Fig. 1). In contrast, when the cells were cultured in the presence of excess thymidine or aphidicolin for 7 days, they accumulated early to middle in S-phase of the cell cycle^4^.

**Figure 3:**
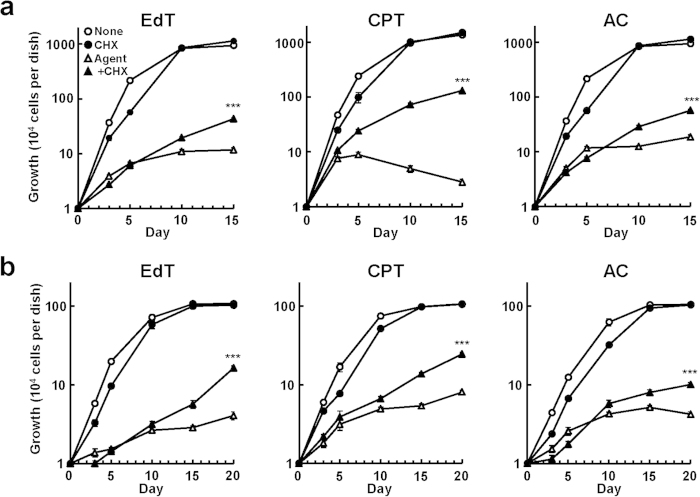
Effect of cycloheximide on growth of human cells cultured with inducers of senescence. (**a**) HeLa were cultured as in Fig. 2, and cell numbers were counted at intervals (n = 4). (**b**) TIG-7 cells were cultured as in Fig. 2, and processed as in (**a**) (n = 4). Bars denote means with a standard error of the mean (SEM). ***P < 0.001 in comparison with the cells added with the agent alone.

Cells continued to grow uniformly, and did not show any sign of senescence when they were cultured in the presence of both cycloheximide and inducers (Figs 2e,f and 3). When HeLa cells were cultured with camptothecin together with cycloheximide, the cell cycle distribution of the cells exhibited a pattern similar to that of normally growing cells (Supplementary Fig. 1). In the presence of the inducers, however, addition of cycloheximide did not allow for a normal growth rate in any of these cases. Therefore, the toxic effects of the inducers themselves do not seem to be eliminated by cycloheximide.

Low concentrations of cycloheximide suppressed senescence features induced by various agents other than the above inducers. These include bleomycin, hydrogen peroxide, paraquat, surfactants, and high salt conditions (Supplementary Fig. 2) besides other DNA damaging agents shown in this study. The inducers of senescence showed differences in their degrees to be suppressed by cycloheximide. This is inevitable since the senescence inducers used have different side effects other than those in delaying DNA replication. Interestingly, senescence like phenomena induced by 5-bromodeoxyuridine^3^ was also suppressed by low concentrations of cycloheximide (Supplementary Fig. 2). To date, cycloheximide seems to suppress senescence features induced by all inducers of senescence and is irrespective of cell-types (Supplementary Figs 2 and 3).

### Characterization of properties associated with cell swelling

**Figure 4:**
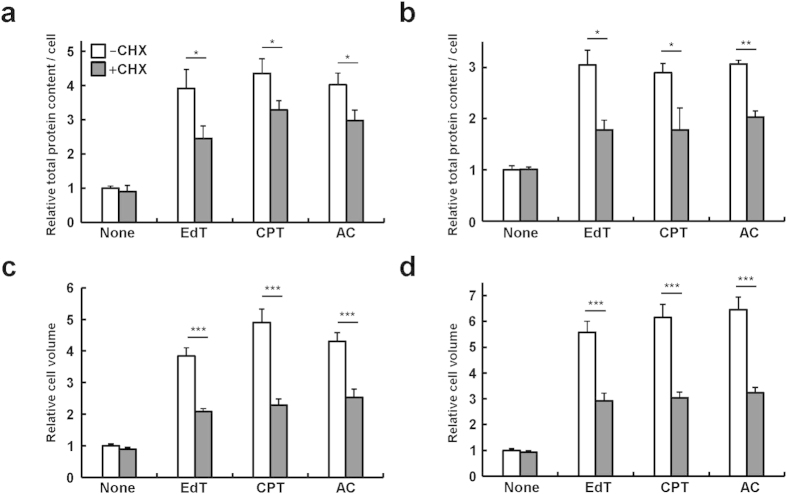
Effect of cycloheximide on protein content and cell volume in human cells. (**a**) HeLa cells were cultured for 7 days as in Fig. 2, and total protein content per cell (n = 3-7) were determined. (**b**) TIG-7 cells were cultured for 7 days as in Fig. 2, and processed as in (**a**) (n = 3). (**c**) Mean volume of HeLa cells (n = 50) were determined. (**d**) Mean volume of TIG-7 cells (n = 50) were determined, and processed as in (**c**). Bars denote means with a standard error of the mean (SEM). *P < 0.05, **P < 0.01 and ***P < 0.001 in comparison between -CHX and + CHX.

Then we examined several characteristics associated with unbalanced growth in HeLa and TIG-7 cells. First, we determined macromolecular synthesis when the cells were cultured under various conditions. Upon addition of excess thymidine, camptothecin, or aphidicolin, total protein content per cell increased by 3–5 folds after 7 days in both cell types (Fig. 4a,b). RNA content per cell increased by approximately 2–3 folds under the same conditions (Supplementary Fig. 4b). Addition of cycloheximide decreased the protein levels by 25-40% in all cases (Fig. 4a,b). Cycloheximide did not significantly affect DNA, RNA, and protein content per cell under normal culture conditions (Fig. 4a,b and Supplementary Fig. 4). The levels of decrease in the protein content were not marked in consistent with use of the low concentration of cycloheximide. The decreases in the protein content correlated well with the degrees to which senescence was suppressed. We then examined cell volumes under the same conditions. Cells were harvested by trypsinization, and spherical cell volumes were measured for 50 cells. Upon addition of excess thymidine, camptothecin, or aphidicolin, cell volumes increased by 4–7 folds after culture for 7 days (Fig. 4c,d). Simultaneous addition of cycloheximide decreased such swollen cell volumes to almost normal levels in all the cases (Fig. 4c,d). Therefore, partial reduction in excess protein content per cell was found to be very effective in reducing cell volumes.

**Figure 5:**
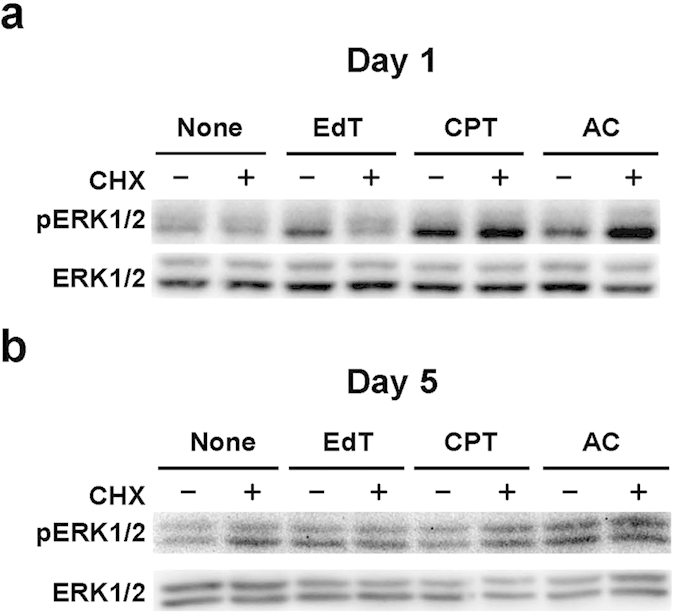
Western blot analysis of ERK1/2 in HeLa cells. (**a**) Cells were cultured in the presence of the agents indicated for 1 day, and cell extracts were subjected to western blot analysis using specific antibodies to total and phosphorylated ERK1/2 as described previously^44^. None, no addition; CHX, 0.15 μM cycloheximide; EdT, 2 mM excess thymidine; CPT, 7.5 nM camptothecin; AC, 0.3 μM aphidicolin. (**b**) Cells were cultured for 5 days, and processed as in (**a**). The full-length images are shown in Supplementary Fig. 7.

In our previous studies, ERK1/2 of the MAP kinase family was activated by treatment with various agents or DNA damages in HeLa and TIG-7 cells^4^,^12^. An inhibitor of ERK1/2 significantly prevented cell swelling induced by excess thymidine or aphidicolin in HeLa cells, although addition of the inhibitors of p38 and JNK besides that of ERK1/2 was necessary to do so in TIG-7 cells^4^,^12^. In these cells, cell volume was returned to a normal level although cellular protein levels were not significantly reduced. Thus, we examined whether cycloheximide can affect phosphorylated ERK1/2 levels in HeLa cells. Upon addition of excess thymidine, camptothecin, or aphidicolin to the cells, the levels of pERK1/2 increased after 1 and 5 days depending on the inducers. Simultaneous addition of cycloheximide did not consistently change the levels, suggesting that the effect of cycloheximide to suppress senescence is not mediated by pERK1/2 (Fig. 5).

### Effect of cycloheximide on replicative cellular senescence

**Figure 6:**
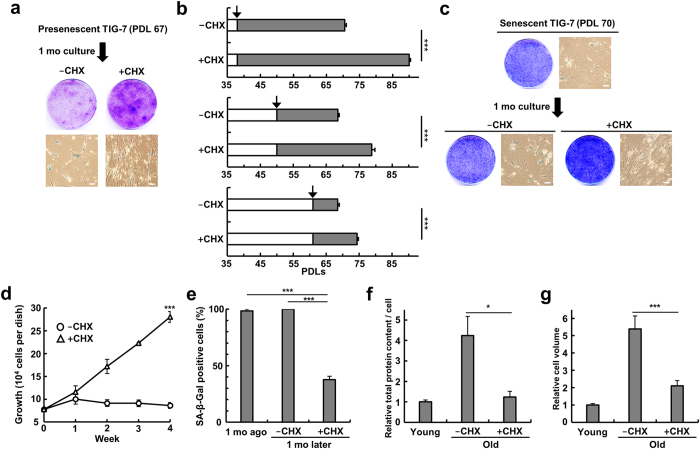
Effect of cycloheximide on replicative senescence in normal human fibroblasts. (**a**) TIG-7 cells at 67 PDLs were cultured for 1 month to form colonies in the presence and absence of 0.075 μM cycloheximide. Colonies were stained with the senescence-associated ß-galactosidase and Coomassie Brilliant Blue. (**b**) TIG-7 cells at various PDLs were cultured by serial passages in the presence and absence of 0.075 μM cycloheximide to determine the maximum lifespan. Arrows indicate the time of addition of cycloheximide (n = 4). (**c**) TIG-7 cells at fully senescent state were cultured in the presence and absence of 0.075 μM cycloheximide for 1 month, and the cells were stained with the senescence-associated ß-galactosidase and Coomassie Brilliant Blue. (**d**) Fully senescent TIG-7 cells were cultured as in (**c**), and cell growth was monitored (n = 3). (**e**) Numbers of TIG-7 cells stained with the senescence-associated ß-galactosidase were calculated (n = 3) as in (**c**). ***P < 0.001 in comparison with the control cells. (**f**) Fully senescent TIG-7 cells were cultured for 1 month as in (**c**), and total protein content per cell was determined (n = 3). (**g**) Mean cell volume for the cells used for (**f**) (n = 50) was determined. Bars denote means with a standard error of the mean (SEM). *P < 0.05, ***P < 0.001 in comparison between -CHX and + CHX. Scale bars, 100 μm.

We examined whether restriction of protein synthesis can extend lifespan of normal human fibroblasts under normal culture conditions. Replicative senescence occurs during serial passages of normal human cells under conventional culture conditions. We used TIG-7 cells at various population-doubling levels (PDLs) and continued to culture in the presence and absence of low concentrations of cycloheximide. When pre-senescent cells (67 PDLs) were used, cycloheximide stimulated their growth and allowed to form large colonies (Fig. 6a). When younger cells (35–50 PDLs) were used, cycloheximide extended their lifespans by 15–20 PDLs (Fig. 6b). These results clearly demonstrate that restriction of protein synthesis can markedly extend lifespan of normal human cells.

Then we designed one challenging experiment. We prepared senescent TIG-7 cells (70 PDLs), which were no longer able to divide. Cycloheximide was added to these cells, and cell culture was continued with periodical changing of medium. Such cells gradually recommenced growth with a very long doubling-time. By one month after addition of cycloheximide, almost all of the cells began to grow, and ß-galactosidase-positive cells were markedly reduced (Fig. 6c,d,e). Several large colonies were observed due to clonal variation of normal cells in a given population. Further, the increased protein content per cell and cell volume in the old cells returned to an almost normal level upon addition of cycloheximide (Fig. 6f,g). These results suggest that replicatively senescent cells have potential to grow when placed under appropriate conditions.

### Effect of cycloheximide on longevity in *C. elegans*

**Figure 7:**
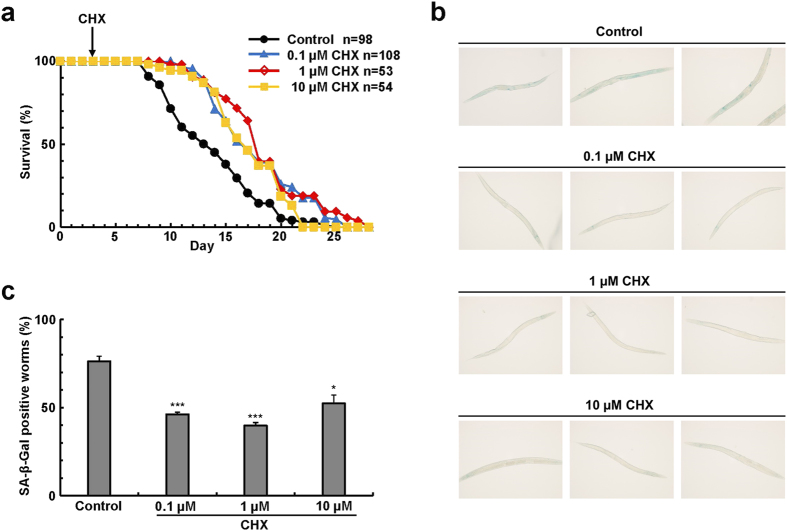
Effect of cycloheximide on longevity in *C. elegans*. (**a**) Indicated numbers of adult N2 worms were placed on NGM agarose medium containing various concentrations (0.1 to 10 μM) of cycloheximide. At intervals the worms were picked up and inoculated on new plates and viable worms were counted every day. Three independent experiments gave similar results. (**b**) L1 worms were cultured for 11 days as in (**a**), and stained with the senescence-associated ß-galactosidase. (**c**) Numbers of worms whose body was totally stained with the senescence-associated ß-galactosidase were counted (n = 3) as in (**b**). Bars denote means with a standard error of the mean (SEM). *P < 0.05, ***P < 0.001 in comparison with the control worms.

Finally, we tried to examine the effect of cycloheximide on longevity with an animal model. We used the nematode *C. elegans*, which is widely used for aging researches as a model system for higher animals^23^. Adult worms were prepared and serially passaged on agar medium containing various concentrations of cycloheximide. We then counted viable and dead worms every day to determine longevity of the worms under protein-restricted conditions. Consequently, the longevity of the nematode was found to markedly increase dependent on the concentrations of cycloheximide (Fig. 7a and Supplementary Table 1). In naturally aged worms, ß-galactosidase-positive cells are shown to increase^24^. We therefore examined whether cycloheximide can reduce ß-galactosidase-positive cells in the aged worms. Addition of a low concentration of cycloheximide clearly decreased the intensity of staining images of the cells (Fig. 7b,c). We measured protein content in the worms, and found that addition of cycloheximide slightly but significantly decrease the protein content (Supplementary Fig. 5).

## Discussion

To date, various approaches have been taken to extend replicative lifespan in normal human cells, including addition of antioxidants to the medium^25^, functional inactivation of p53 and/or RB^26^, and enforced expression of the catalytic subunit of telomerase^27^, *etc*. These approaches are shown to be effective to various degrees in delaying replicative lifespan probably due to the degree in DNA damage arising from oxidative stress or blocking signaling pathways capable of responding to oxidative stress. These approaches, however, are not effective in suppressing induced senescence in normal and immortalized cell types. In sharp contrast, mild restriction of cytoplasmic protein synthesis with low concentrations of cycloheximide was shown to suppress all aspects of senescence in virtually any systems of cellular senescence. It extended replicative lifespan of normal human fibroblasts greater than the above approaches. Interestingly, it allowed replicatively senescent human fibroblasts to significantly grow. These results show that the phenomenon of replicative senescence may be a reversible and meaningful state of the cell cycle.

Our results also suggest that imbalance in major macromolecules (DNA, RNA, and protein) has a major role in senescence at the cellular and organismal levels. In relation to this study, rapamycin^28^ and methionine restriction^22^ are shown to extended lifespan in several animal models, although their mechanisms remain largely unknown. Methionine restriction is suggested to be due to improved stress tolerance in rodents^29^ because it modulated the mitochondrial protein synthesis and the mitochondrial respiratory chain assembly, but did not affect cytoplasmic protein synthesis in human diploid fibroblasts^22^. Dietary restriction of amino acids other than methionine also prevented oxidative damage during aging^30^. Lifespan extension by conditions that inhibit mRNA translation is also observed in *C. elegans*^31^,^32^. Taken together, the mechanisms underlying these effects are distinct from that of low concentrations of cycloheximide.

How does cytoplasmic protein restriction suppress cellular senescence ? We described earlier that unbalanced growth leads to cell swelling and then nuclear swelling irrespective of induces or cell types^6^. Nuclear swelling is thought to mechanically disintegrate the structure of the nuclear envelope to which the heterochromatin was bound. Since the heterochromatin is normally condensed to silence the so-called senescence-associated genes that are preferentially located on it^7^,^8^, the collapse of the nuclear envelope due to mechanical or physiological causes is thought to decondense it, leading to expression of such genes. In fact, destabilization of nucleosome positioning by changing DNA conformation is suggested to lead to disintegration of the nuclear envelope and expression of the silenced genes^33^,^34^.

In eukaryotic cells with dynamic and fragile cell structures, a harmonized balance of macromolecules seems extremely important to maintain cell integrity that constitutes a fundamental substratum for life. Under the conditions unfavorable for growth, this balance tends to lose its equilibrium. To date, proliferating mammalian cells do not have a strict feedback regulatory mechanism by which retardation in DNA replication restricts protein synthesis, although a week feedback control is shown to work in HeLa cells^35^. After searching for various agents or treatments, very subtle limitation of cytoplasmic protein synthesis was found to be effective in correcting imbalance of macromolecules during unbalanced growth. For unbalanced growth to occur, several specific signaling pathways seem to be involved. We have already found that the cell-cycle checkpoint mechanisms composed of ERK1/2 and Chk1/2 have a major role in stabilizing cells during unbalanced growth (Ikuru Kudo *et al.* manuscript in preparation).

Practically, mild protein restriction may be beneficial with no side effect to reduce dysfunctions due to ageing in humans. It is desirable to find a substitute for cycloheximide from natural resources, which can be orally ingested to exert a holistic health promoting effect, to reduce excess or unnecessary protein synthesis *in vivo*. In the human body, a vast amount of proteins are degraded to amino acids via peptides and reused all the time. Accumulation of harmful peptides, which is inevitable due to uncontrolled cleavage of proteins by various proteases, causes senescence *in vitro*^36^ and various diseases *in vivo*^37^. Therefore, protein restriction may be also beneficial in decreasing production of harmful peptides and facilitating excretion of them.

## Methods

### Cell culture

Normal human fibroblast strain TIG-7 and Cervical tumor cell line HeLa were obtained from the Japanese Collection of Research Bioresources (JCRB). Human colon carcinoma cell line HCT116 and its p21-negative mutant HCT116 p21^−/−^ were obtained from B. Vogelstein of Howard Hughes Medical Institute. Mouse embryonic line NIH3T3, human fibroblastic cell line SUSM-1, human fibrosarcoma line HT1080, Chinese hamster ovary line CHO, and mouse mammary ascites line FM3A were obtained from JCRB. TIG-7 cells were cultured at 37 °C in plastic Petri dishes (Thermo Scientific, Nunc) containing Dulbecco’s modified Eagle’s medium (DMEM) supplemented with 10% fetal calf serum under 5% CO_2_ and 95% humidity. HeLa cells were cultured in ES medium supplemented with 5% calf serum. HCT116 cells were cultured in ES medium supplemented with 10% fetal calf serum. NIH3T3, SUSM-1, HT1080 and CHO cells were cultured in DMEM supplemented with 5% calf serum. FM3A cells were cultured in ES medium supplemented with 2% calf serum.

To assay colony formation, cells were fixed and stained with Coomassie Brilliant Blue, and subjected to photography. To monitor cell growth, cells were harvested by trypsinization and cell numbers were counted with a hematocytometer.

### ß-Galactosidase assay

Assay in human cells was performed as described previously^3^. Cells were fixed in 2% formaldehyde/ 0.2% glutaraldehyde at room temperature for 5 min, and incubated at 37 °C with a fresh staining solution [1 mg/ml of 5-bromo-4-chloro-3-indolyl ß-D-galactoside, 40 mM citric acid-sodium phosphate (pH 6.0), 5 mM potassium ferricyanide, 5 mM potassium ferrocyanide, 150 mM NaCl, and 2 mM MgC1_2_].

### Determination of macromolecules

Cells were suspended in 10 mM Tris-EDTA (pH 7.5), and disrupted by sonication for a total of 1 min on ice. After centrifugation of the cell lysate at 15,000 rpm at 4 °C for 5 min, the resulting supernatant was used as a cell extract. For measurement of DNA and RNA, nucleic acid was extracted with phenol-chloroform, precipitated with ethanol, and resolved in 10 mM Tris-EDTA (pH 7.5). After treatment with DNase or RNase, DNA and RNA were specifically determined by Burton’s diphenylamine reaction method^38^ and Schneider’s orcinol reaction method^39^, respectively. Protein was determined with Bio-Rad Protein Assay Kit (Bio-Rad). Macromolecular contents were normalized by cell number. To measure protein content in nematode *C. elegans*, worms were cultured axenically, collected, disrupted by sonication, and the resulting cell lysate was used for determination.

### Determination of cell volume

Cells were harvested by trypsinization and suspended in phosphate-buffered saline. Pictures were taken and diameters were measured for 50 cells to calculate their relative cell volumes.

### Western blot analysis

Cells were suspended in 30 mM Tris-HCl (pH 8.0) containing 50 mM vanadate and 2 nM okadaic acid, and disrupted by sonication for a total of 1 min on ice. After centrifugation of the cell lysate at 15,000 rpm at 4 °C for 5 min, the resulting supernatant was used as a cell extract. Protein was determined for this sample as described above. Cell extracts were subjected to 12% SDS polyacrylamide gel electrophoresis, blotted onto a PVDF-membrane and probed with specific antibodies against pERK1/2, ERK1/2 (pT202/pY204) (MAP kinase Activation Sampler Kit, BD Biosciences). The specific mouse antibodies were detected with a chemiluminescence detection kit (Boehringer Mannheim). Chemiluminescence was exposed to an X-ray film as described previously^3^.

### Determination of longevity in nematode *C. elegans*

Longevity of nematodes was determined as described previously^22^. N2 worms of *C. elegans* were synchronized with alkaline hypochlorite solution treatment and inoculated on lawns of *E. coli* OP50 strain grown on NGM medium contained in 60 mm plastic dishes. When the worms reached L3 larvae, FdUrd (60 μM) was added to the medium. Adult worms were picked up and transferred to new 30 mm dishes containing the bacteria and FdUrd every 2–3 days, and both viable and dead worms were counted every day.

### ß-Galactosidase assay in the nematode *C. elegans*

N2 worms were synchronized at L1 state, and transferred to agar plates containing various concentrations of cycloheximide. After culture for 11 days, the worms were collected, and stained with ß-galactosidase activity as described previously^23^.

## Additional Information

**How to cite this article**: Takauji, Y. *et al.* Restriction of protein synthesis abolishes senescence features at cellular and organismal levels. *Sci. Rep.* **6**, 18722; doi: 10.1038/srep18722 (2016).

## Supplementary Material

Supplementary Information
