# Supplementary material for: Restriction of protein synthesis abolishes senescence features at cellular and organismal levels
Source: Sci Rep. 2016 Jan 5;6:18722. doi: 10.1038/srep18722 (PMC4700526; doi:10.1038/srep18722)
Supplement: Supplementary Information [file srep18722-s1.pdf]

Supplementary Information for

Restriction of protein synthesis abolishes senescence features at cellular and organismal levels

Yuki Takauji[1], Takumi Wada[1], Asuka Takeda[1], Ikuru Kudoh[1], Kensuke Miki[1,2], Michihiko Fujii[1] and Dai Ayusawa[1,2]*

[1] *Graduate School of Nanobioscience, Yokohama City University, 22-2 Seto, Kanazawa-ku, Yokohama, Kanagawa 236-0027, Japan*
[2]*Ichiban Life Corporation, 1-1-7 Horai-cho, Naka-ku, Yokohama, Kanagawa 231-0048, Japan*

*To whom correspondence should be addressed.
Email address: dayusawa@yokohama-cu.ac.jp

Supplementary Table 1. Effect of cycloheximide on longevity in *C. elegans.*

Experiment		Conc. (μM)	N	Mean lifespan (day)	Maximum lifespan (day)	*P*-value	% Increase
I	Control	0	50	15.7	19		
	CHX	3.5	50	17.2	25	<0.05	10.1
		35	50	17.8	23	<0.001	13.7
		350	50	15.2	19	n.s.	-3.1
II	Control	0	130	16.0	27		
	CHX	0.1	80	18.0	27	<0.001	12.8
		1	80	19.4	28	<0.001	19.1
		10	87	18.5	29	<0.001	13.1
III	Control	0	98	12.9	24		
	CHX	0.1	108	16.9	25	<0.001	30.7
		1	53	17.6	27	<0.001	36.1
		10	54	16.1	21	<0.001	24.8

Lifespan of N2 worms of *C. elegans* was determined on NGM agarose medium containing various concentrations of cycloheximide as described in Methods. In the first and third experiments, cycloheximide was added to adult worms, and in the second experiment L1 worms. Mean lifespan is the day when 50% of the worms died. Maximum lifespan is the day when the last worm died. *P*-value is compared with control. % Increase was calculated by $(A-B)/B$ x100, where A and B are the mean lifespans in the presence and absence of cycloheximide, respectively.

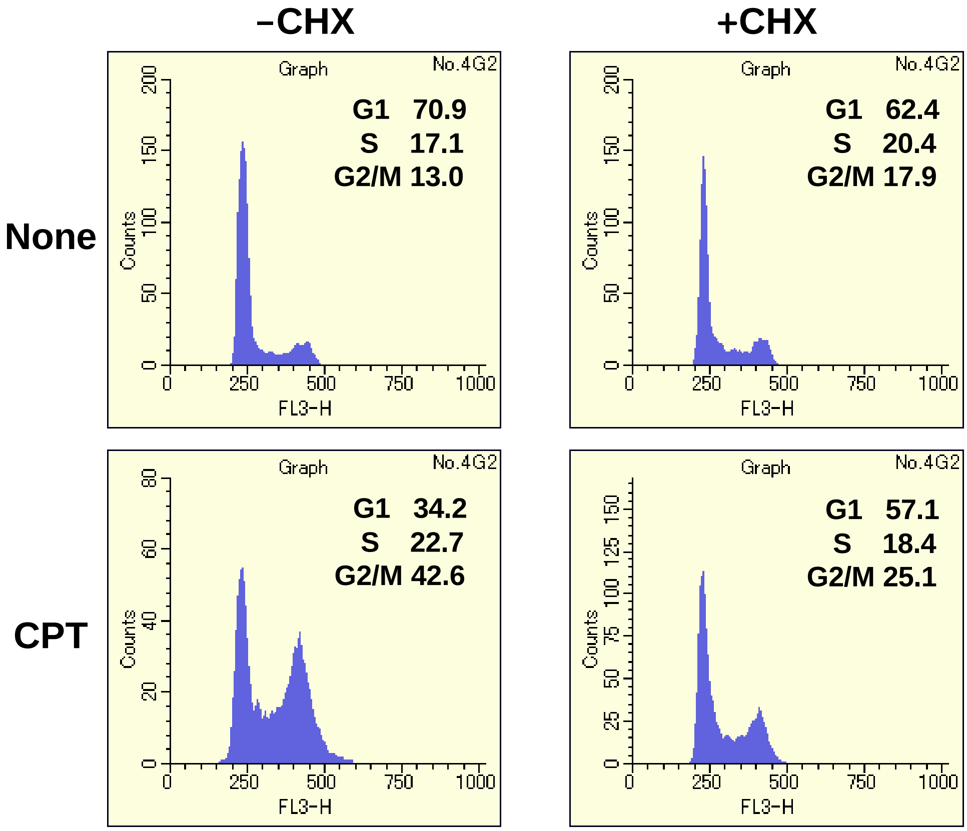

Supplementary Figure 1. Effect of cycloheximide on cell cycle distribution of HeLa cells. cultured with camptothecin in the presence and absence of cycloheximide.

Cells were cultured for 7 days as in Fig. 2, stained with propidium iodine, and subjected to flow cytometry as described [4]. Distributions of cells (%) at each phase of the cell cycle are shown inside of each panel.

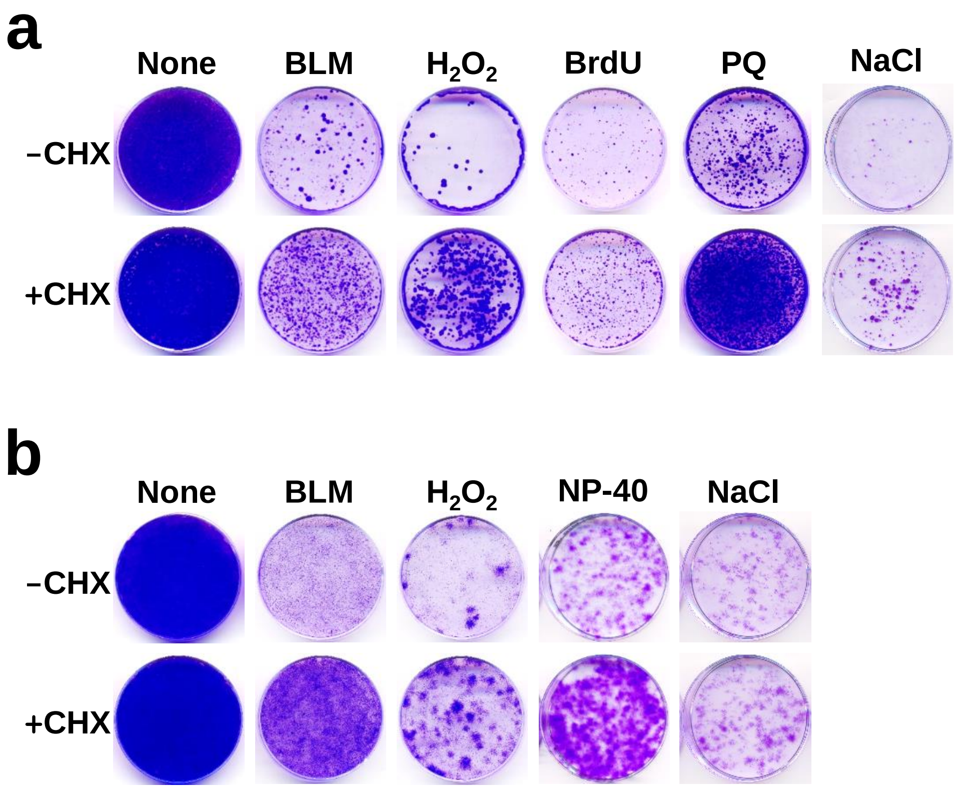

Supplementary Figure 2. Effect of cycloheximide on the loss of colony forming ability induced by various agents in human cells.

(a) HeLa cells were cultured in the presence of the agent indicated for 2 weeks, and colonies formed were stained with Coomassie Brilliant Blue as described in Methods. None, no addition; CHX, 0.15 μM cycloheximide; BML, 1 μM bleomycin; H_2O_2, 70 μM hydrogen peroxide; BrdU, 50 μM 5-bromo-2'-deoxyuridine; PQ, 50 μM paraquat; NaCl, 100 mM sodium chloride. (b) TIG-7 cells were cultured for 3 weeks, and processed as in (a). None, no addition; CHX, 0.15 μM cycloheximide; BML, 0.05 μM bleomycin; H_2O_2, 50 μM hydrogen peroxide; NaCl, 100 mM sodium chloride; NP-40, 15 μM octylphenoxypolyethoxyethanol.

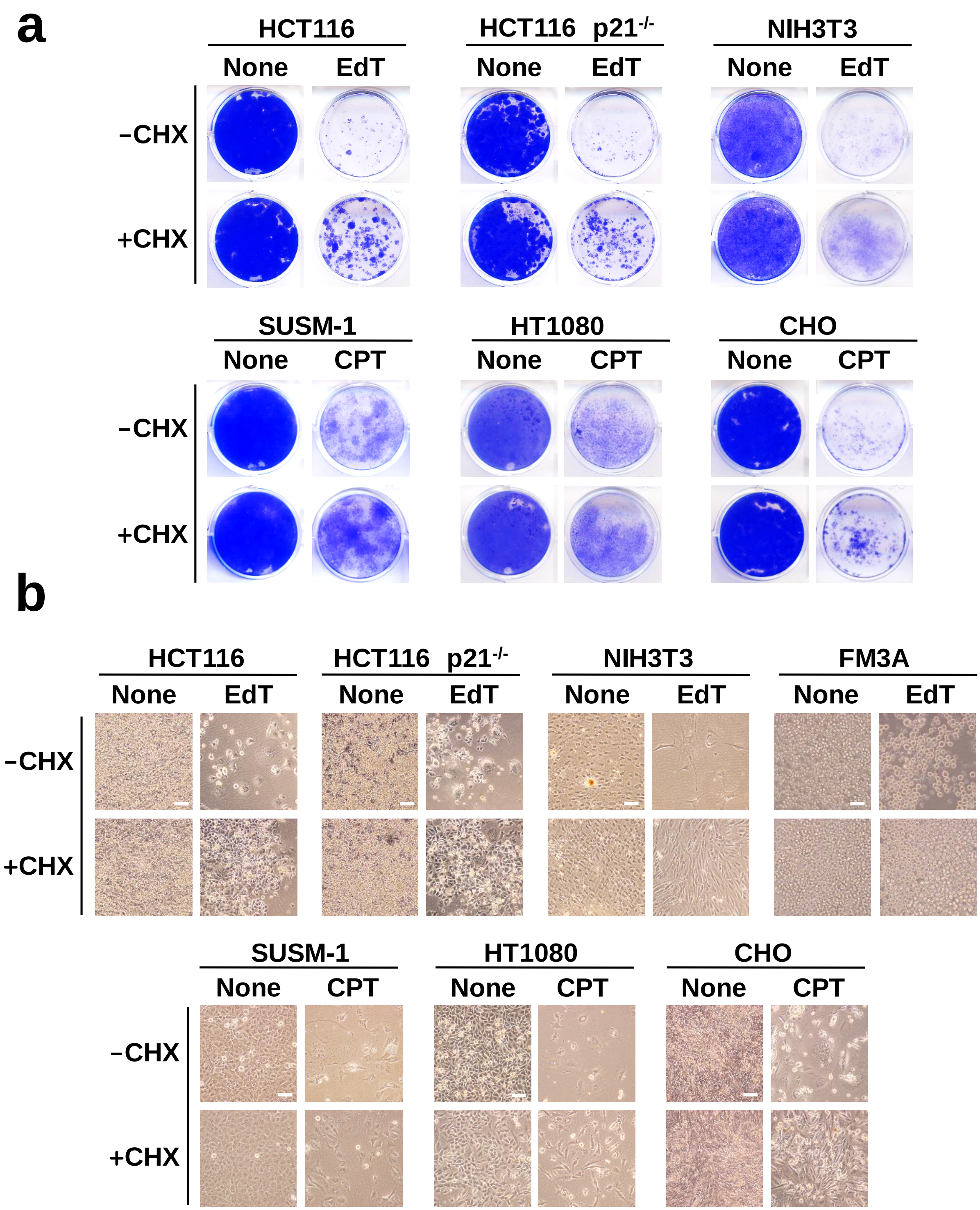

Supplementary Figure 3. Effect of cycloheximide on senescence features induced by excess thymidine or camptothecin in various cell types.

(a) Various cell types were cultured in the presence of the agent indicated for 2 weeks, and colonies formed were stained with Coomassie Brilliant Blue. None, no addition; CHX, cycloheximide (0.075 µM for HCT116, HCT116 p21$^{-/-}$ and HT1080, 0.0325 µM for NHI3T3, FM3A, SUSM-1 and CHO); EdT, excess thymidine (2 mM for HCT116, 1 mM for HTC116 p21$^{-/-}$ and NIH3T3, 0.5 mM for FM3A); CPT, camptothecin (4.5 nM for SUSM-1, 5.5 nM for HT1080 and 20 nM for CHO). (b) Cells were cultured as in (a), and subjected to photography. Scale bars, 100 µm.

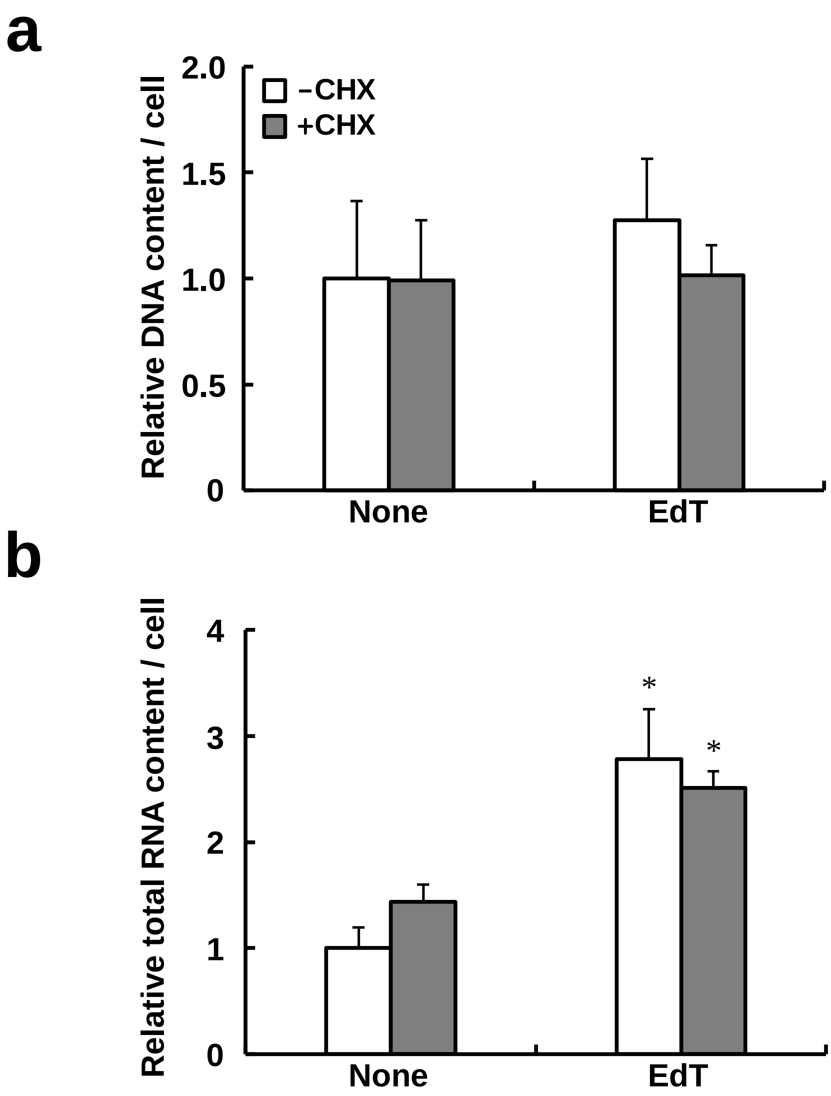

Supplementary Figure 4. Effect of excess thymidine and cycloheximide on DNA and RNA content per cell in HeLa cells.

(a) Cells were cultured for 7 days as in Fig. 1, and DNA content per cell was determined (n=3) as described in Methods (b) RNA content per cell was determined (n=3) for the same cells as described in Methods. . Bars denote means with a standard error (SEM). *P<0.05 in comparison with the control cells (None).

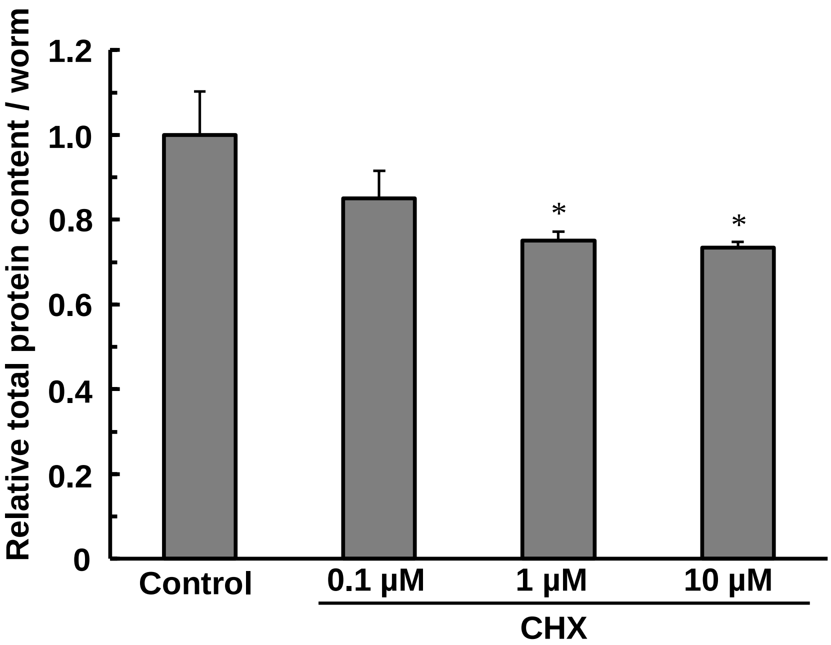

Supplementary Figure 5. Effect of cycloheximide on protein content in *C. elegans.*
N2 worms were synchronized at L1 state and cultured axenically for 4 days in liquid medium containing 30 mg/mL yeast extract, 30 mg/mL soy peptone, 10 mg/mL glucose, 0.5 mg/mL hemoglobin, 10 µg/mL cholesterol, 10 µg/mL streptomycin and 100 µg/mL ampicillin. After culture for 4 days, worms were harvested and total protein content per worm was determined as described above for the cultured cell (n=5). Bars denote means with a standard error of the mean (SEM). *P<0.05 in comparison with the control worms.

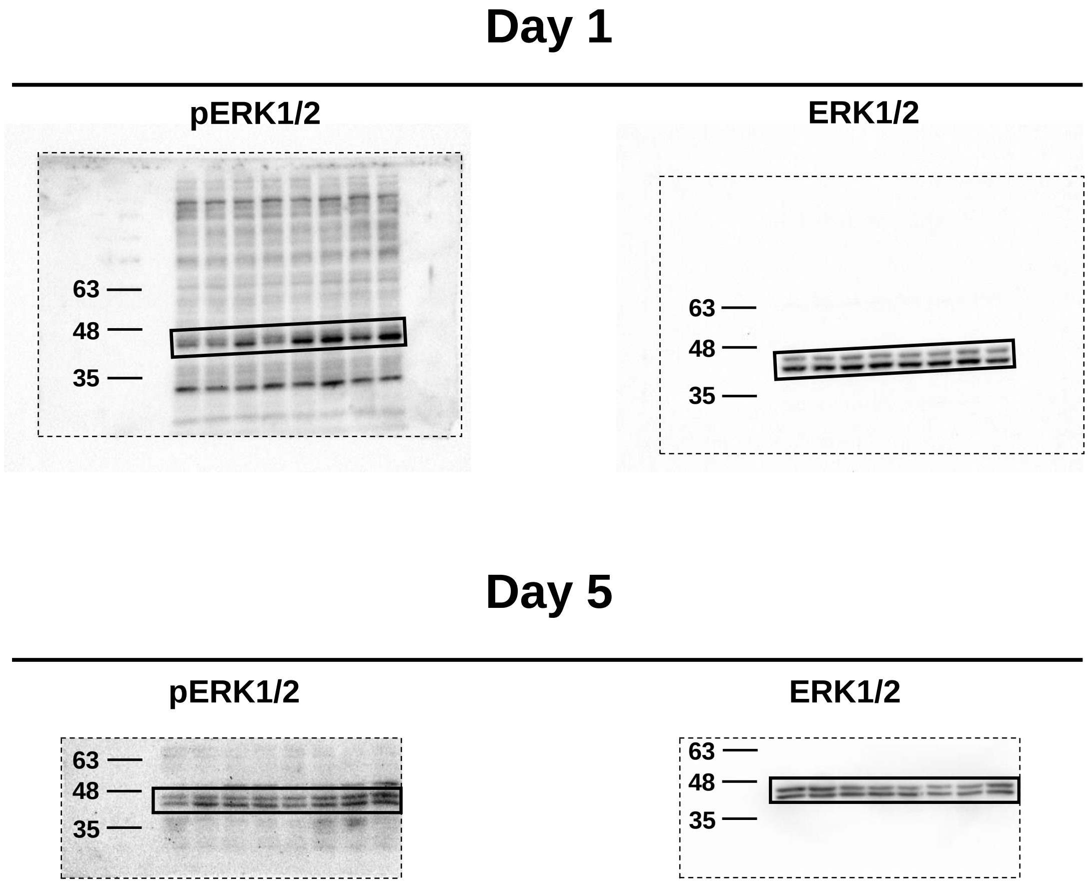

Supplementary Figure 6. Full-length images of Fig. 5.
The origin of these figures is identical with Fig. 5 showing western blotting with pERK1/2 or ERK1/2 antibody. Cropping lines were indicated with squares. The dotted broken lines indicate the margins of the gels.
